# Identification of vital prognostic genes related to tumor microenvironment in pheochromocytoma and paraganglioma based on weighted gene co-expression network analysis

**DOI:** 10.18632/aging.202754

**Published:** 2021-03-26

**Authors:** Chun-Xian Chen, Dong-Ning Chen, Xiong-Lin Sun, Zhi-Bin Ke, Fei Lin, Hang Chen, Xuan Tao, Fei Huang, Yong Wei, Ning Xu

**Affiliations:** 1 Department of General Medicine, The First Affiliated Hospital of Fujian Medical University, Fuzhou 350005, China; 2 Department of Urology, The First Affiliated Hospital of Fujian Medical University, Fuzhou 350005, China; 3 Department of Pathology, The First Affiliated Hospital of Fujian Medical University, Fuzhou 350005, China; 4 Central Lab, The First Affiliated Hospital of Fujian Medical University, Fuzhou 350005, China

**Keywords:** pheochromocytoma and paraganglioma, tumor microenvironment, prognostic, WGCNA, bioinformatic methods

## Abstract

Pheochromocytoma and paraganglioma (PCPG) is a rare neuroendocrine tumor. This study aims to identify vital prognostic genes which were associated with PCPG tumor microenvironment (TME). We downloaded transcriptome data of PCPG from TCGA database and calculated the immune scores and stromal scores by using the ESTIMATE algorithm. DEGs related to TMB were then identified. We conducted WGCNA to further extract the TME-related modules. GO, KEGG pathway analysis, and PPI network were performed. Survival analysis was conducted to identify the hub genes associated with the prognosis of PCPG. A total of 150 PCPG samples were included in this study. We obtained 1507 and 2067 DEGs based on immune scores and stromal scores, respectively. WGCNA analysis identified the red module and brown module were correlated with immune sores while the turquoise module and red module were significantly associated with stromal scores. Functional enrichments analysis revealed that 307 TME-related genes were correlated with the inflammation or immune response. Survival analysis showed that three TME-relate genes (ADGRE1, CCL18, and LILRA6) were associated with PCPG prognosis. These three hub genes including ADGRE1, CCL18, and LILRA6 might be involved in the progression of PCPG and could serve as potential biomarkers and novel therapeutic targets.

## INTRODUCTION

Pheochromocytoma and paraganglioma (PCPG) is a rare neuroendocrine tumor that produces catecholamines (CA) and originates from adrenal medulla or extra-adrenal ganglia [1]. It refers to pheochromocytoma as the neoplasm arises from the adrenal gland, whereas it is termed paraganglioma when the tumor originates from extra-adrenal tissues. Tumoral secretion of catecholamines accounts for characteristic clinical symptoms particularly episodic or sustained hypertension, palpitations, headache, and diaphoresis. Also, PCPG can lead to disorders in insulin metabolism, cardiovascular morbidity, and even mortality [2]. Most PCPGs are benign in their clinical presentation, however, have the potential risk of malignant transformation [3, 4]. Patients with metastatic PCPG have a 5-year survival rate ranging from 40-77% [3]. At present, surgery is the main way to treat non-metastatic PCPG, yet there has been no standardized therapeutic regimen for metastatic PCPG [4]. Further investigation into the molecular mechanism using bioinformatics methods would provide novel insight into diagnosis and prognosis of PCPG.

Tumor microenvironment (TME) is defined as the cellular environment where the tumor cells are located, and is composed of different cell types including immune cells, stromal cells and endothelial cells etc [5]. In recent years, TME has been regarded as a critical factor in tumor progression and metastasis. However, the impact of TME on PCPG remains unclear. Immune cells and stromal cells are the major components of non-tumor cells in TME, and play an essential role in tumor diagnosing and prognostic predicting. Accumulated evidence has shown that tumor gene expression profile can quantify the immune infiltrating landscape of tumors.

The ESTIMATE (Estimation of STromal and Immune cells in MAlignant Tumor tissues using Expression data) algorithm proposed by Yoshihara et al. [6] has been applied to estimate the proportion of infiltrating stromal and immune cells in malignancy by using the expression profile. Previous studies have proved that the ESTIMATE algorithm was effective for calculating stromal scores, immune scores and tumor purity, thus providing a useful method for predicting the prognosis of patients using gene expression data [6, 7]. However, there were no studies available exploring tumor microenvironment of PCPG utilizing ESTIMATE algorithm or high throughput data.

The weighted gene co-expression network analysis (WGCNA) has been widely used in biology and medical research and offers an effective approach to detect a group of genes with similar expression patterns as well as their relevant biological processes and pathways [8, 9]. In this way, modules are defined as the clusters of highly correlated genes.

In the present study, we extracted the expression data of PCPG cohorts from TCGA database and then applied the ESTIMATE algorithm and the WGCNA method to perform a comprehensive analysis of tumor microenvironment-related genes for the first time. Our results indicated that the tumor microenvironment associated genes could affect the clinical prognosis of PCPG patients, suggesting that it might provide a basis for development of new prognostic biomarkers and therapeutic targets.

## RESULTS

### Evaluation of immune/stromal scores and DEGs screening

**Figure 1:**
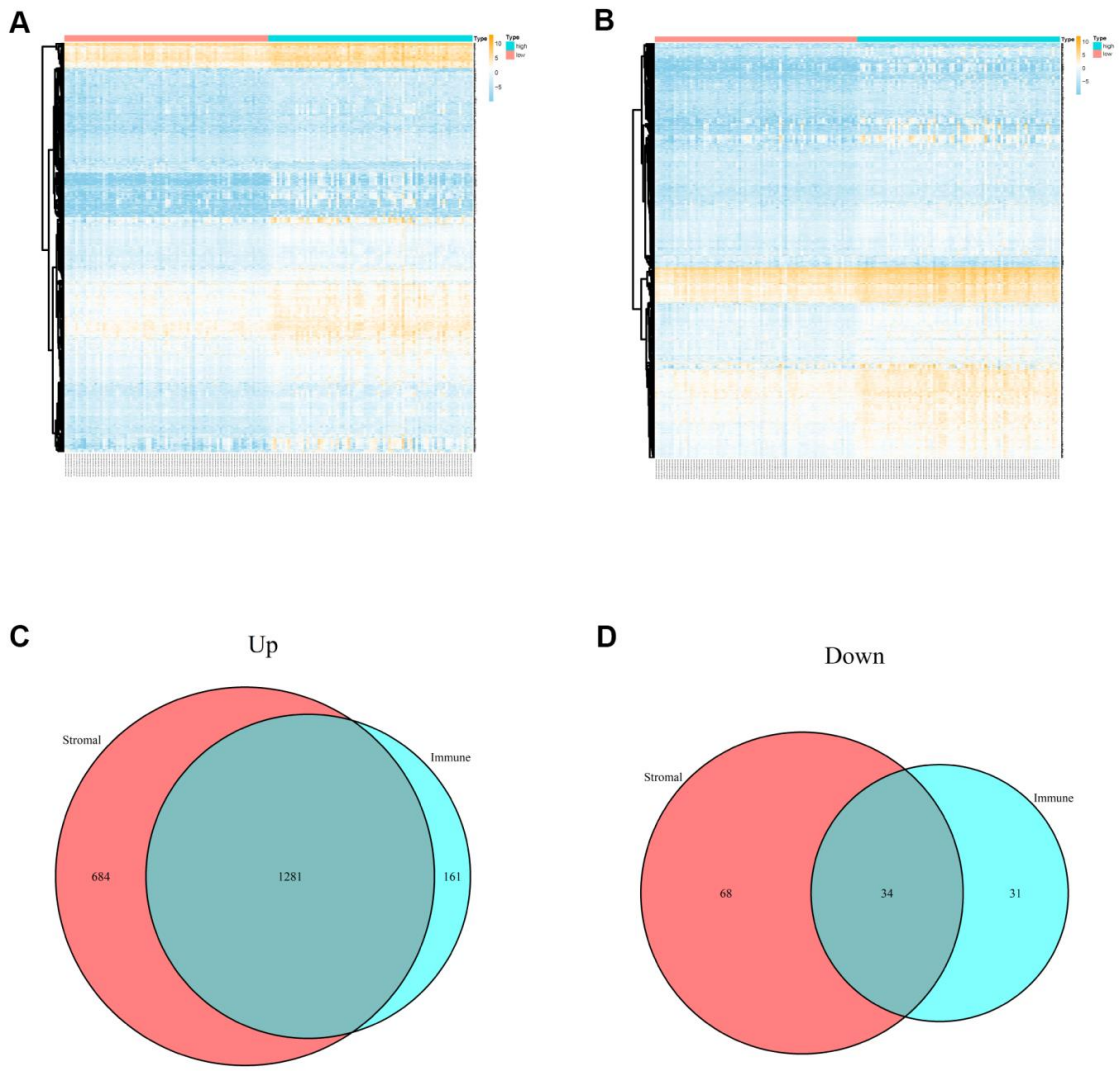
**Differentially expressed genes of immune scores and stromal scores.** (**A**) The heatmap of top 100 DEGs by comparing high scores with low scores of immune scores. (**B**) The heatmap of top 100 DEGs by comparing high scores with low scores of stromal scores. (**C**, **D**) Venn plots displaying co-upregulated and co-downregulated DEGs respectively.

A total of 150 cases of PCPG from TCGA database were included in the present study and 3 normal cases were used. Based on the ESTIMATE algorithm, the stromal scores were ranged from -2002.755 to 1445.792, and the immune scores were ranged from -1503.612 to 2140.047. We divided all PCPG cases into high stromal score group and low stromal score group according to median value. Analogously, all PCPG patients were also classified into high immune score group and low immune score group according to median value. Based on stromal scores, 2067 DEGs related to stromal scores (including 1965 up-regulated DEGs and 102 down-regulated DEGs) were screened. Besides, 1507 DEGs related to immune sores (including 1442 up-regulated DEGs and 341 down-regulated DEGs) were obtained based on immune score (Figure 1A, 1B). The Venn plots showed that there were a total of 1281 co-upregulation genes and 34 co-downregulation genes (Figure 1C, 1D).

### Construction of weighted gene co-expression modules

**Figure 2:**
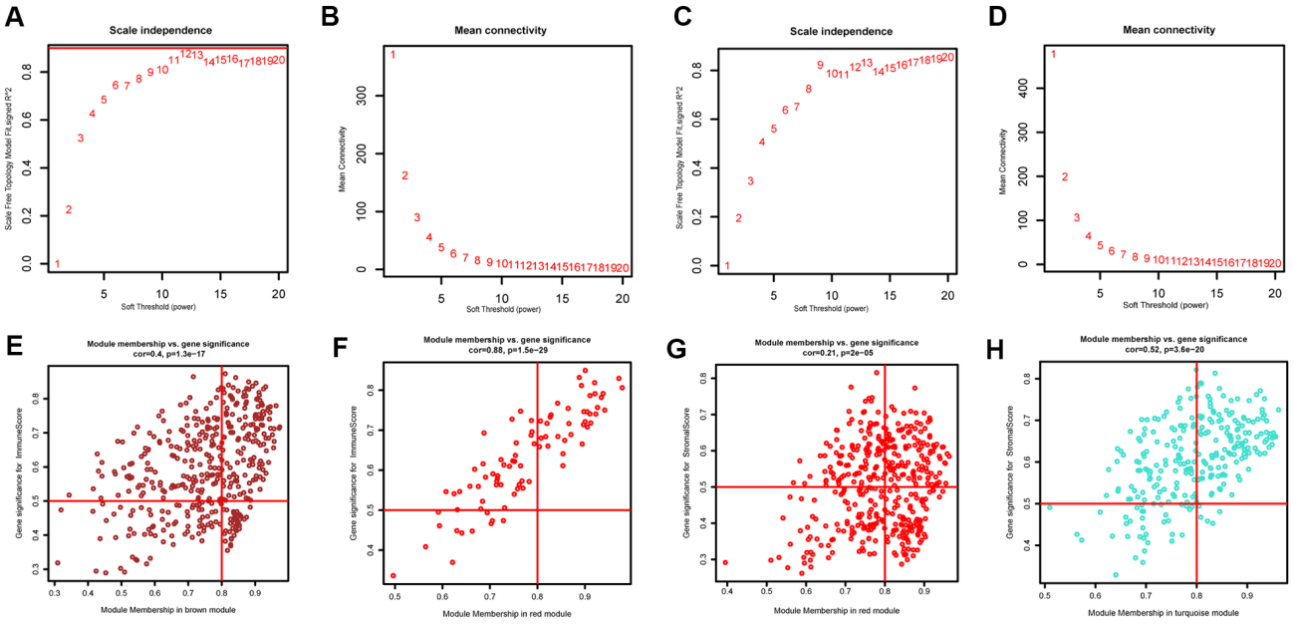
**Determination of soft-thresholding power and scatter plot of module eigengenes in WGCNA.** (**A**, **C**) Analysis of the scale-free fit index for various soft-thresholding powers. (**B**, **D**) Analysis of the mean connectivity for various soft-thresholding powers. (**E**, **F**) Scatter plot of module eigengenes in key modules that were highly correlated with immune sores. (**G**, **H**) Scatter plot of module eigengenes in key modules that were highly correlated with stromal sores.

**Figure 3:**
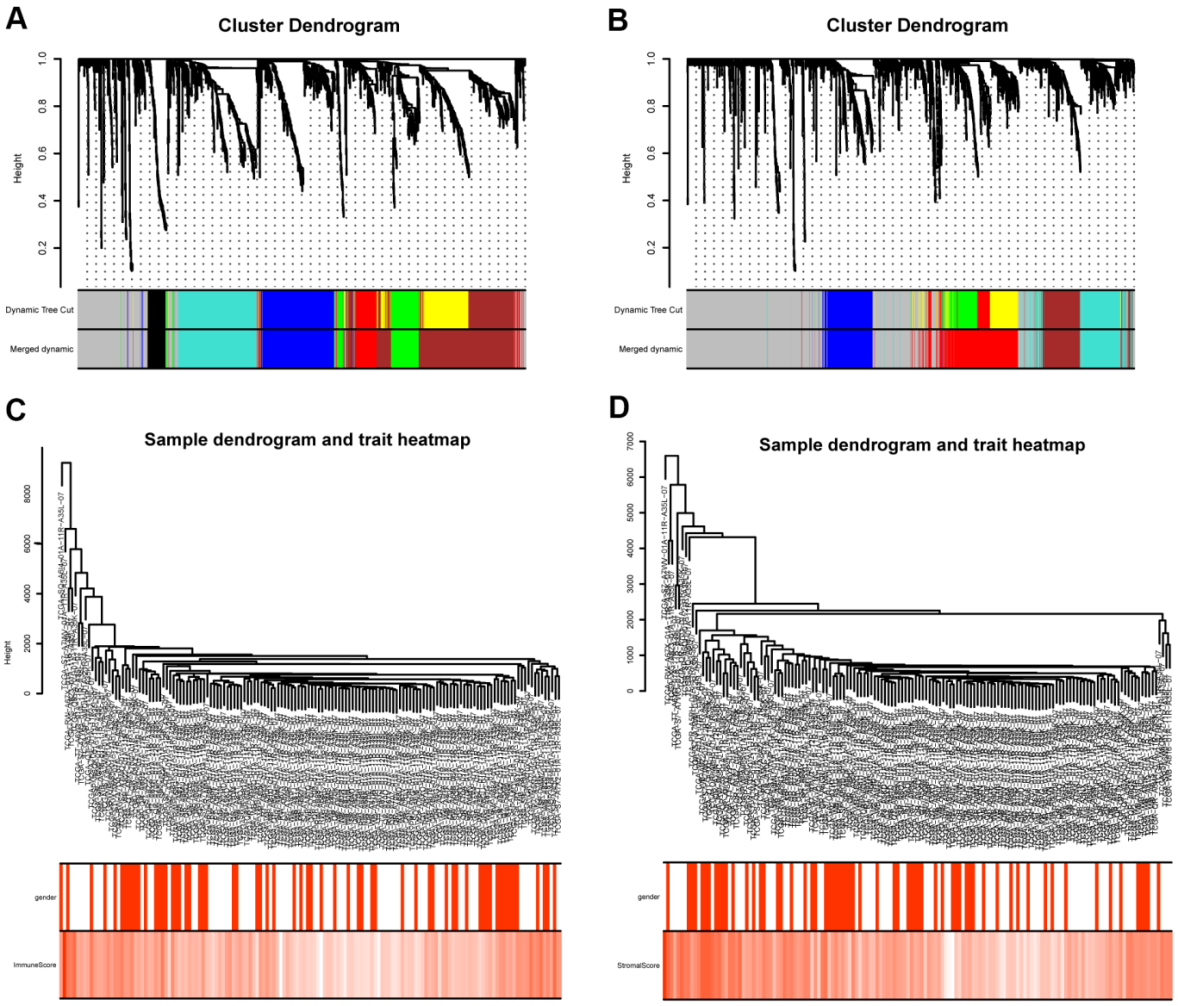
**Gene coexpression module analysis.** (**A**) Gene dendrogram and identified coexpression modules of DEGs between high- and low- immune score groups. (**B**) Gene dendrogram and identified coexpression modules of DEGs between high- and low- stromal score groups. (**C**) Sample dendrogram and trait heatmap of DEGs between high- and low- immune score groups. (**D**) Sample dendrogram and trait heatmap of DEGs between high- and low- stromal score groups.

**Figure 4:**
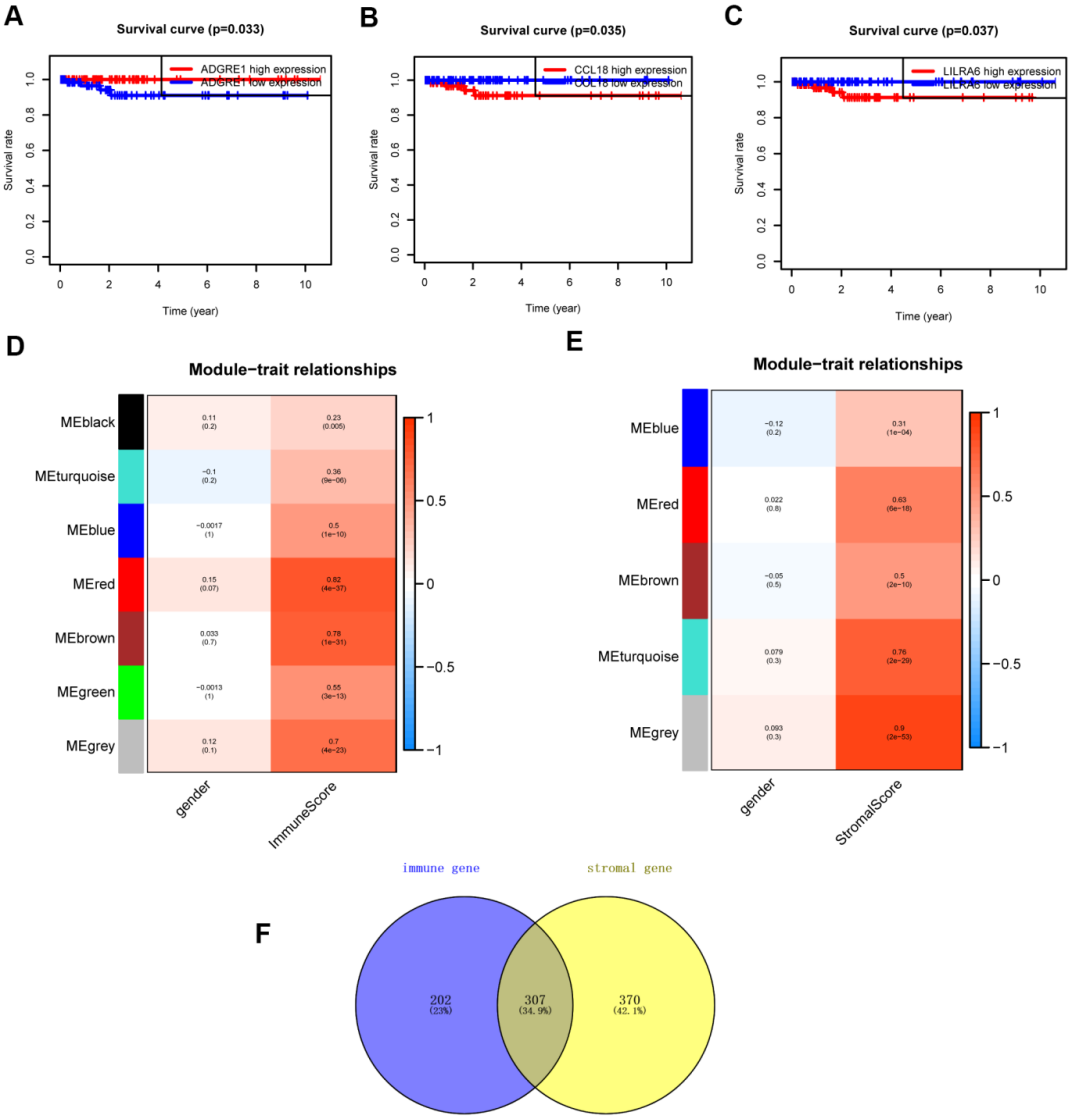
**Survival analysis and module-trait relationship analysis.** (**A**–**C**) Overall survival between patients with high and low expression of the three hub genes. (**D**) Heatmap of the correlation between module eigengenes and immune score. (**E**) Heatmap of the correlation between module eigengenes and stromal score. (**F**) Venn plots displaying the intersection genes.

The “WGCNA” package in R was applied to put the DEGs with similar expression patterns into modules by average linkage clustering. The best soft-thresholding parameter was set at 8 (scale-free *R*^2^ = 0.85) to guarantee a scale-free network (Figure 2A–2D). A sample dendrogram was then constructed based on the similarity between the samples and the clinical characteristics of each sample (Figure 3C, 3D). Finally, 7 gene co-expression modules of immune scores-related genes and 5 gene co-expression modules of stromal scores-related genes were identified, respectively (Figure 3A, 3B). Module-trait relationship analysis showed that Module Eigengene (ME) of the red module and brown module were highly correlated with immune sores while the turquoise module and red module were significantly associated with stromal scores (Figures 2E–2H, 4D, 4E). In this way, we obtained 509 immune sores-related genes and 677 stromal scores-related genes from those key modules. 307 intersection genes were selected for further analysis (Figure 4F).

### Functional enrichment analyses

**Figure 5:**
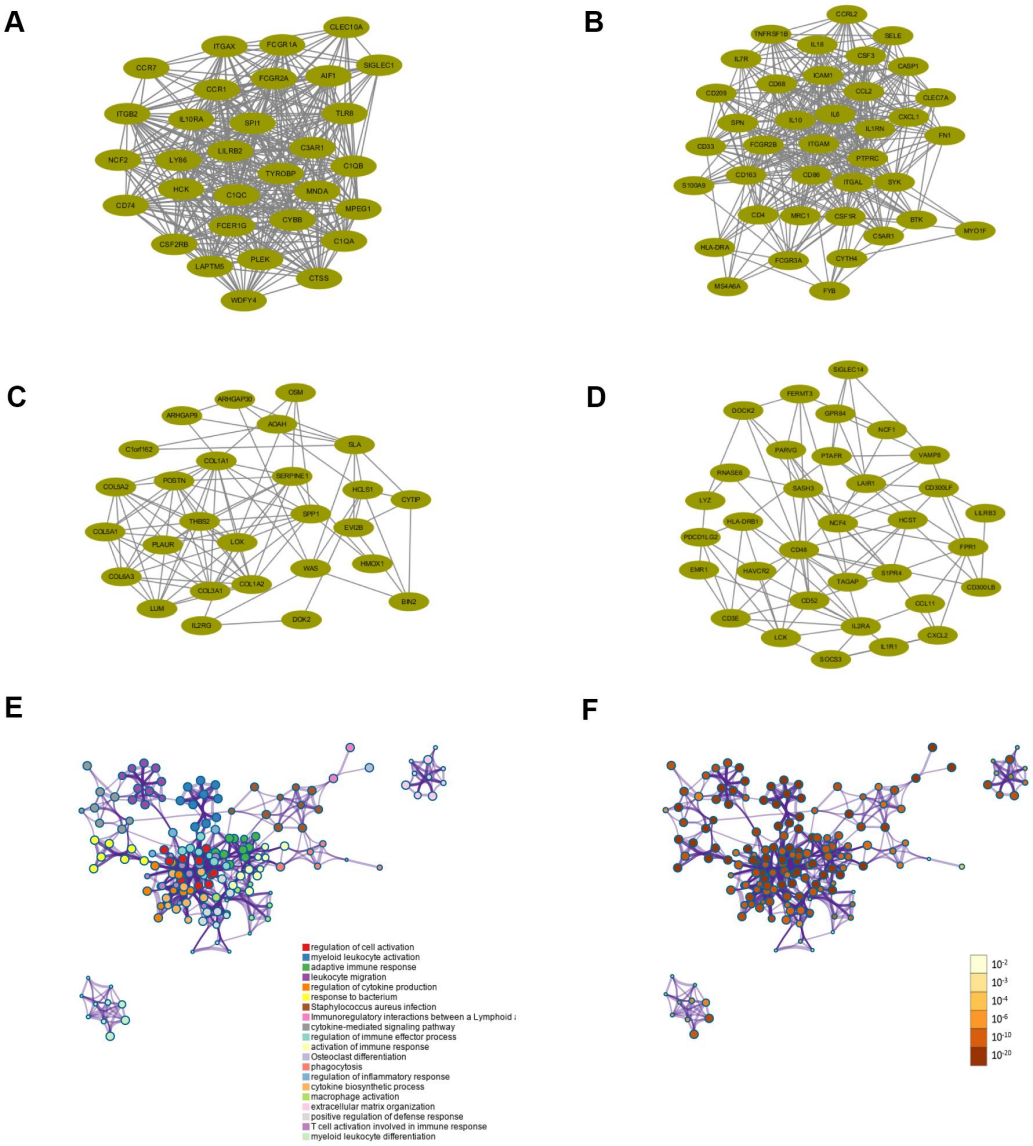
**Functional enrichment analysis and construction of PPI network.** (**A**–**D**) PPI network of four core modules constructed using STRING. (**E**) Functional enrichment analysis for the 307 intersection genes. (**F**) P-value of each gene in the network.

Functional enrichment analyses were conducted to investigate the biological processes and pathways relevant to these 307 genes using Metascape databases. The top 20 enriched biological processes and pathways were demonstrated in Figure 5 and Table 1, including regulation of cell activation, myeloid leukocyte activation, adaptive immune response, leukocyte migration, regulation of cytokine production, response to bacterium, Staphylococcus aureus infection, etc. The results showed that biological processes and pathways were mainly related to the inflammation or immune response.

**Table 1:** Functional enrichment analysis for the intersection genes.

**Category**	**Term**	**Count**	**%**	**Log10 (P)**	**Log10 (q)**
GO Biological Processes	regulation of cell activation	69	23.08	-44.26	-39.94
GO Biological Processes	myeloid leukocyte activation	67	22.41	-41.05	-37.2
GO Biological Processes	adaptive immune response	67	22.41	-39.4	-35.69
GO Biological Processes	leukocyte migration	55	18.39	-34.94	-31.53
GO Biological Processes	regulation of cytokine production	64	21.4	-34.24	-30.87
GO Biological Processes	response to bacterium	59	19.73	-30.36	-27.12
KEGG Pathway	Staphylococcus aureus infection	23	7.69	-28.9	-25.81
Reactome Gene Sets	Immunoregulatory interactions between a Lymphoid and a non-Lymphoid cell	30	10.03	-28.6	-25.56
GO Biological Processes	cytokine-mediated signaling pathway	59	19.73	-28.31	-25.33
GO Biological Processes	regulation of immune effector process	47	15.72	-28.21	-25.25
GO Biological Processes	activation of immune response	56	18.73	-27.54	-24.6
KEGG Pathway	Osteoclast differentiation	28	9.36	-26.11	-23.28
GO Biological Processes	phagocytosis	41	13.71	-26.08	-23.27
GO Biological Processes	regulation of inflammatory response	44	14.72	-23.66	-21.06
GO Biological Processes	cytokine biosynthetic process	21	7.02	-17.23	-14.82
GO Biological Processes	macrophage activation	19	6.35	-17.11	-14.71
GO Biological Processes	extracellular matrix organization	32	10.7	-17.1	-14.7
GO Biological Processes	positive regulation of defense response	36	12.04	-16.28	-13.91
GO Biological Processes	T cell activation involved in immune response	18	6.02	-15.07	-12.76
GO Biological Processes	myeloid leukocyte differentiation	23	7.69	-14.77	-12.48

### PPI network, GO and KEGG analysis

All intersection genes were mapped into STRING database and the PPI networks were built using Cytoscape software. The plug-in MCODE screened four top core modules, which were shown in Figure 5A–5D. These PPI networks of core modules related to tumor microenvironment might be of great importance for PCPG development and progression. Enrichment analyses of GO and KEGG were conducted to assess the functions of the genes in the four identified modules, respectively. The most enriched GO and KEGG terms preserved in each module were displayed in Tables 2, 3, respectively. For the first module (module 1), positive regulation of cytokine production and cytokine receptor activity were the most significant enrichment in biological process and molecular functions, respectively. In the meantime, KEGG analysis revealed that genes in this module were enriched in complement and coagulation cascades pathway and Osteoclast differentiation pathway. For the second module (module 2), cellular response to biotic stimulus, secretory granule membrane and cytokine receptor binding were the most significant enrichment in biological process, molecular function and cellular component, respectively. For the third module (module 3), the GO analysis indicated that these genes were mainly involved in extracellular matrix structural constituent. Protein digestion and absorption, ECM-receptor interaction, and PI3K-Akt signaling pathway were the most significantly enriched pathways. The genes in the fourth module (module 4) were primarily enriched in positive regulation of leukocyte cell-cell adhesion, Osteoclast differentiation pathway and Th17 cell differentiation pathway.

**Table 2:** GO enrichment for the genes in the key modules of PCPG.

**Module**	**Term**	**Count**	***P*-value**
Module 1-BP	GO:0001819: positive regulation of cytokine production	10	1.57E-09
	GO:0019882: antigen processing and presentation	8	1.90E-09
	GO:0043312: neutrophil degranulation	10	2.41E-09
Module 1-CC	GO:0030667: secretory granule membrane	8	1.45E-08
	GO:0070821: tertiary granule membrane	5	9.54E-08
	GO:0070820: tertiary granule	6	1.87E-07
Module 1-MF	GO:0004896: cytokine receptor activity	5	4.53E-07
	GO:0019864: IgG binding	3	6.47E-07
Module 2-BP	GO:0071216: cellular response to biotic stimulus	17	2.09E-23
	GO:0007159: leukocyte cell-cell adhesion	16	4.69E-19
	GO:0002237: response to molecule of bacterial origin	16	6.23E-19
Module 2-CC	GO:0009897: external side of plasma membrane	12	3.93E-12
Module 2-MF	GO:0005126: cytokine receptor binding	9	3.24E-09
	GO:0005125: cytokine activity	7	2.09E-07
Module 3-BP	GO:0030199: collagen fibril organization	7	7.13E-13
	GO:0030198: extracellular matrix organization	11	8.92E-13
	GO:0043062: extracellular structure organization	11	3.94E-12
Module 3-CC	GO:0005583: fibrillar collagen trimer	6	1.67E-15
	GO:0098643: banded collagen fibril	6	1.67E-15
Module 3-MF	GO:0005201: extracellular matrix structural constituent	9	6.88E-13
	GO:0030020: extracellular matrix structural constituent conferring tensile strength	6	1.81E-11
Module 4-BP	GO:1903039: positive regulation of leukocyte cell-cell adhesion	7	7.06E-08
	GO:0007159: leukocyte cell-cell adhesion	8	7.48E-08
	GO:0043312: neutrophil degranulation	9	8.25E-08
	GO:0002283: neutrophil activation involved in immune response	9	8.69E-08
Module 4-CC	GO:0070820: tertiary granule	7	6.84E-09
Module 4-MF	GO:0016176: superoxide-generating NADPH oxidase activator activity	2	0.000115733422069724

**Table 3:** KEGG pathway enrichment for the genes in the key modules of PCPG.

**Module**	**Term**	**Count**	***P*-value**
Module 1	hsa05150: Staphylococcus aureus infection	7	8.23E-09
	hsa05152: Tuberculosis	8	2.97E-08
	hsa04610: Complement and coagulation cascades	6	1.36E-07
Module 2	hsa05144: Malaria	8	2.03E-11
	hsa05152: Tuberculosis	11	9.72E-11
	hsa05323: Rheumatoid arthritis	8	3.40E-09
Module 3	hsa04974: Protein digestion and absorption	6	1.71E-08
	hsa04512: ECM-receptor interaction	5	5.61E-07
	hsa04151: PI3K-Akt signaling pathway	7	2.48E-06
Module 4	hsa04380: Osteoclast differentiation	6	8.85E-07
	hsa04659: Th17 cell differentiation	5	8.43E-06

### Survival analysis of intersection genes

Additional survival analysis was conducted on the 307 intersection genes to evaluate their effects on the survival of PCPG. Finally, three hub genes (ADGRE1, CCL18, and LILRA6) (Figure 4) were identified to be associated with overall survival time (p < 0.05). We found that the high levels of CCL18 and LILRA6 were significantly correlated with poor survival outcomes, while the high levels of ADGRE1 was associated with longer overall survival.

### Validation of expression levels of hub genes

**Figure 6:**
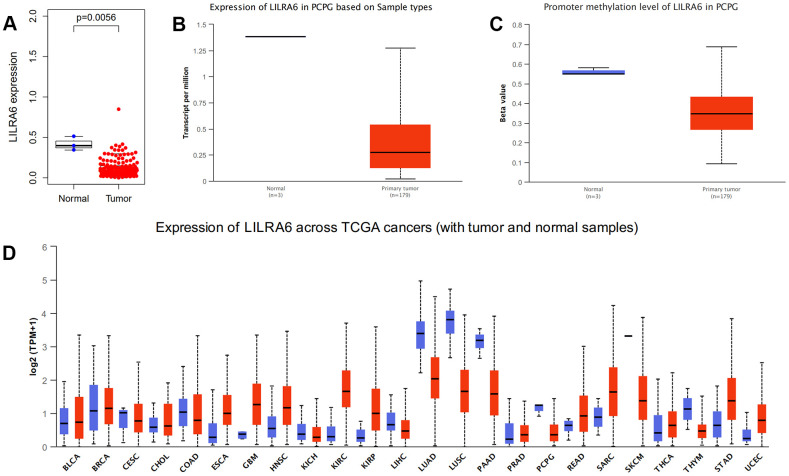
**Validation of expression levels of the LILRA6.** Different expression levels of the LILRA6 between normal and tumor tissue in TCGA (**A**) and ULCAN (**B**) database. The promoter methylation level of LILRA6 between normal and tumor tissue (**C**). Pan-cancers analysis of LILRA6 (**D**).

We further validated the differential expression of ADGRE1, CCL18, and LILRA6 between PCPG tissue and normal tissue in TCGA database and ULCAN database. The results showed that only LILRA6 were differently expressed between tumor and normal tissues. The promoter methylation level of LILRA6 was also significantly lower tumor tissue compared with normal tissue. Besides, pan-cancers analysis showed that LILRA6 was not specific to PCPG. However, there were few molecular markers of PCPG at present and LILRA6 might be a vital marker in PCPG immune microenvironment. (Figure 6).

## DISCUSSION

Increasing evidence indicates that the tumor microenvironment plays a critical role in tumor progression and metastasis [5, 10]. Tumor development is highly related to the TME, and any alterations of tumor microenvironment may influence the clinical outcomes of malignancies. However, the effect of TME differs in different types of cancer. The correlation between TME and the PCPG prognosis remains poorly understood. In this study, we calculated the stromal scores and immune scores using the ESTIMATE algorithm to estimate the level of infiltrating stromal and immune cells in PCPG. A total of 2067 DEGs related to stromal scores and 1507 DEGs related to immune sores were obtained from comparison of low- and high- level groups. Then, the WCGNA analysis was conducted and 307 genes related to PCPG tumor microenvironment were identified. Finally, three tumor microenvironment-related genes (ADGRE1, CCL18, and LILRA6) significantly associated with PCPG prognosis were obtained by survival analysis.

Functional enrichment analysis was conducted and PPI networks were built to further investigate the biological functions. The results revealed that the biological processes and pathways were mainly related to the inflammation or immune response. Likewise, the GO and KEGG analyses showed that the top GO terms and pathways enriched in the four core modules were also significantly associated with the inflammation and immune response. According to the previous studies, it was generally acknowledged that systemic inflammation played a central role in tumorigenesis and cancer progression. Several reports have shown that fever of unknown origin and systemic inflammatory syndrome were associated with IL-6 producing pheochromocytoma, which tended to have a larger tumor volume as well as an elevated risk of pheochromocytoma multisystem crisis [11, 12]. Furthermore, the inflammation has long been considered to be closely associated with the pathogenesis of RCC [13]. A variety of systemic inflammatory indexes have been identified to exhibit a predictive value for RCC, including NLR, lymphocyte-to-monocyte ratio and platelet count [14]. Karin et al. [15, 16] reported that chronic inflammation caused by prolonged infection with a bacterium, parasite, or virus was a main driving force in cancer development. It was found that the persistent inflammatory microenvironment set by HBV and HCV virus infections induced Hepatocellular carcinoma development [17–19]. Similarly, Chronic Helicobacter pylori infection increased the likelihood of mucosa-associated lymphoid tissue cancer and gastric cancer development. Cytokines were the key signaling molecules of communication between cells in the inflammatory tumor-microenvironment. The cytokines released during the persistent infection tended to induce several molecular signaling cascades, ultimately promoting neoplastic processes [20, 21].

CCL18 is a C-C chemokine mainly secreted by M2 type tumor associated macrophages which acts mainly by binding to its corresponding chemokine receptor CCR8 [22]. Emerging evidence indicates that CCL18 serves numerous functions not only closely related to immune and inflammatory modulation, but also involved in cancer progression [23]. Wang et al. [24] reported that CCL18 could promote tumor angiogenesis, repress anti-cancer immune reaction and reshape TME, thus, leading to malignant progression in diverse human cancers. Moreover, another study revealed that CCL18 affected the replicative ability of tumor cells by promoting cell transformation and altering the number of tumor cell chromosomes [25]. High expression CCL18 was tightly related to poor prognosis in various tumor, including ovarian cancer [26], pancreatic ductal adenocarcinoma [27] gallbladder carcinoma [28] and gastric cancer [29] etc. Additionally, CCL18 was expressed not only in cells of the immune system, but also in tumor cells. Liu et al. [22] reported for the first time that CCL18 was up-regulated in bladder cancer (BC) cells, which further promote cell migration, invasion and epithelial-mesenchymal transition (EMT). CCL18 overexpression has also been revealed to be associated with the proliferation, invasiveness, and angiogenesis of oral cancer cells, as well as the TNM stage [30]. However, there was no study investigating the role in PCPG. This study revealed that the high expression of CCL18 was associated with poor prognosis and might be a promising candidate gene affecting the occurrence and development of PCPG.

LILR, also known as the leukocyte Ig-like receptors, are a family of innate immune receptors that finely balance the functions of immune system and dictate their response to infected, stressed, and an aggressive tumor behavior [31]. Members of the LILR family played a vital role in various immune response processes such as cytokine production, DC maturation, and co-stimulatory molecules expression [32, 33]. In a previous study, Jones et al [34]. reported that the paired activating and inhibitory LILRB3 and LILRA6 receptors could shape the local inflammatory responses in epithelial tumors by interacting with a ligand associated with the expression of cytokeratin 8. The balance of signals transmitted by paired LILRB3/LILRA6 receptors might therefore determine whether an immune response was triggered to the necrotic tumor cell, thereby leading to a wider immune response within the tumor microenvironment. In our study, the upregulation of LILRA6 was associated with poor prognosis in PCPG. Therefore, the development of specific LILRA6 inhibitors may provide an attractive target for anti-tumor immunotherapy in further study.

ADGRE1, named EMR1 in the past, is a member of adhesion G protein–coupled receptor family and has been regarded as a specific marker for eosinophils in humans [35]. However, the exact function of ADGRE1 is still unknown. Qi et al. [36] reported that ADGRE1, a down-regulated hub gene, was associated with the alpha fetoprotein (AFP) level in hepatocellular carcinoma (HCC) and may have crucial roles in HCC progression. Waddell et al [37] suggested the hypothesis that ADGRE1 could evolved in the process of immune selection and pathogen recognition, thus, participating in the host defense. Moreover, a previous study showed that afucosylated chimeric antibodies directed against ADGRE1 provided a promising target for immune cell ablation strategies [38]. In this study, we found that ADGRE1 was associated with longer overall survival in PCPG which indicated a protective role in PCPG biogenesis. However, the mechanism of ADGRE1 in PCPG development remains unknown and further research is required to be investigated.

In our study, we found that high expression levels of CCL18 and LILRA6 were significantly correlated with poor survival, while the high levels of ADGRE1 were associated with longer overall survival. Interestingly, the results showed that all of the three hub genes belong to the 1281 upregulated genes. That is to say, the stromal and immune cells seem to be playing a dual role, either as the promoter or suppressor, during PCPG development and progression. To date, several studies have described the dual effect of the tumor stroma in the tumor-host interaction [39, 40]. The stroma cells preferentially served an antitumor role in the tumor microenvironment. With ongoing tumor growth, stromal cells have been shown to promote growth and invasive behavior of tumor cells by secretion of growth factors, neovascularisation and facilitating recycling of anaerobic metabolic products [41]. Along with stromal cells, immune cells infiltrating the tumor tissue were also associated positively or negatively with tumor progression [42]. It was well known that CD8+ T cells were essential for immune defense and cytotoxic anti-tumor immunity. By contrast, CD4+ regulatory T cells suppressed anti-tumor immunity, thereby limiting the long-term efficacy of cancer immunotherapy [43]. Previous studies have reported that an imbalance of immune response may lead to oncogenesis and cancer progression [42]. Therefore, the immunomodulation may play multiple roles in the progression of PCPG and the specific mechanism remains an area of intense study.

There were several limitations in this study. First of all, due to the rather low incidence of PCPG, there were rare additional dataset of PCPG in several most commonly used database. We mainly explored the potential functions and hub genes of tumor immune microenvironment of PCPG based on WGCNA and TCGA database. Secondly, this was a retrospective study and the sample size was limited. Further prospective study is required. Finally, we just showed that these DEGs were correlated with ESTIMATE stromal or immune scores; however, we did not clarify whether these genes were expressed in immune or stromal cells. Whether these genes were expressed in immune or stromal cells or tumor cells required further cytological experiment.

## CONCLUSIONS

In summary, by using a comprehensive bioinformatics analysis, we identified three tumor microenvironment-related genes (ADGRE1, CCL18, and LILRA6) which were closely associated with the prognosis of PCPG. These key genes may act as potential biomarkers and novel therapeutic targets. Further studies are required to analysis and validate the function of the identified genes and pathways *in vitro* and *in vivo*.

## MATERIALS AND METHODS

### Data collection and preprocessing

**Figure 7:**
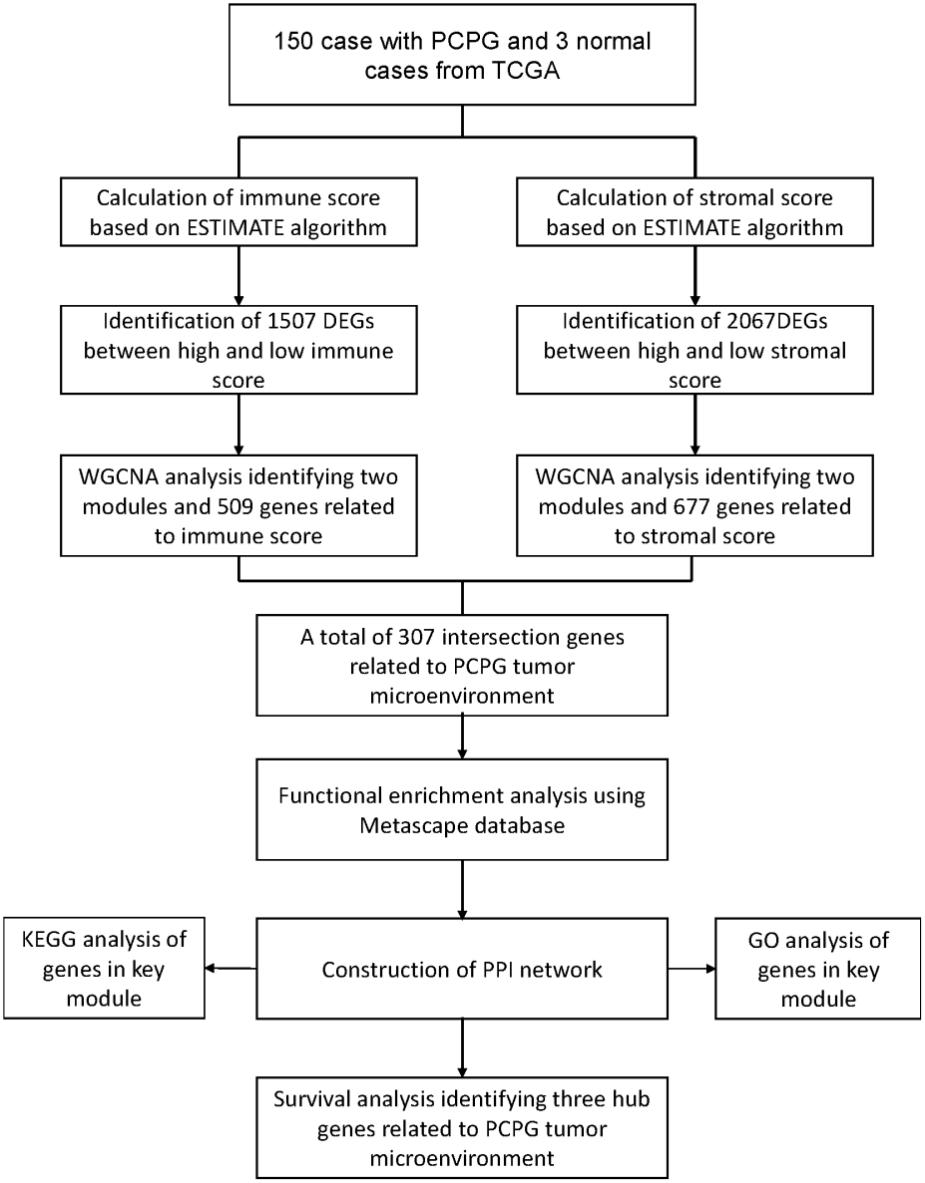
**The flow diagram of this study.**

The overall workflow of the present study was shown in Figure 7. We downloaded the level 3 gene expression profile, including 150 PCPG samples and 3 normal samples, from the cancer genome atlas (TCGA) database (https://portal.gdc.cancer.gov/). The immune scores and stromal scores were then calculated by using the ESTIMATE algorithm accordingly [6].

### Differential gene expression analysis

Enrolled samples were divided into low- and high- level groups according to the median value of scores from the ESTIMATE algorithm. To reveal the correlations between the gene expression profiles and the stromal scores, “limma” package was applied to identify differentially expressed genes (DEGs). The selection criteria for DEGs were as follows: log |fold change (FC)| > 1 and adj. p <0.05. Heatmaps in this study were generated using “pheatmap” R package as described previously [44]. Likewise, we performed the same analysis procedure in the immune scores group.

### Weighted gene co-expression network analysis (WGCNA)

After screening the differentially expressed genes (DEGs), WGCNA was conducted using the “WGCNA” package in R to identify the co-expression modules and significant genes that have similar patterns of expression to each other [45, 8]. The dynamic tree cut method was employed to identify modules. Modules were named using different colors. Highly similar modules were identified by cluster analysis and the merged cutting height of 0.25 was set. Pearson correlation coefficients between modules and clinical traits were calculated, and modules significantly correlated with immune/stromal scores were selected (P < 0.05). The intersection genes in those key modules were then subjected to further analysis and the Venn diagrams were generated with the VennDiagram R package.

### Functional enrichment analysis

Metascape (http://metascape.org/) is an online database providing a comprehensive set of functional annotation tools to determine the biological relevance behind large list of genes [46]. We uploaded the intersection genes to perform GO analysis and pathway enrichment analysis. *P*-value <0.05 was- considered statistically significant.

### Construction of PPI network, GO analysis, and KEGG

All intersection genes were mapped to the Search Tool for the Retrieval of Interacting Genes (STRING) database, which is an online database for constructing protein-protein interaction (PPI) networks [47]. The MCODE plug-in in the Cytoscape software was used to screen the core modules [48]. To reveal the potential functions of significant genes in candidate modules, Gene Ontology (GO) term and Kyoto Encyclopedia of Genes and Genomes (KEGG) pathway enrichment analyses were then performed using R software, with p-value <0.05 as the cutoff value.

### Survival analysis

Survival analysis was performed using the survival R package. The Kaplan-Meier plots was drawn to assess the relationship between intersect genes and overall survival, and the log-rank test was applied to test the significance of the difference between the two. P < 0.05 was considered to be significant.

### Validation of expression levels of hub genes

UALCAN database (http://ualcan.path.uab.edu/) was used to validate the different expression levels, promoter methylation level of the hub genes between PCPG tissues and normal tissues. Additionally, the expression levels of hub genes were also compared with other tumor types to explore whether these genes were specific to PCPG.

### Data availability statement

All data generated or analyzed during the present study was downloaded from the cancer genome atlas (TCGA) database and UALCAN database, and could be available freely from the corresponding author (Ning Xu).

## References

[1] Jha A, de Luna K, Balili CA, Millo C, Paraiso CA, Ling A, Gonzales MK, Viana B, Alrezk R, Adams KT, Tena I, Chen A, Neuzil J, et al. Clinical, Diagnostic, and Treatment Characteristics of SDHA-Related Metastatic Pheochromocytoma and Paraganglioma. Front Oncol. 2019; 9:53. 10.3389/fonc.2019.00053 30854332 PMC6395427

[2] Lenders JW, Eisenhofer G. Update on modern management of pheochromocytoma and paraganglioma. Endocrinol Metab (Seoul). 2017; 32:152–61. 10.3803/EnM.2017.32.2.152 28685506 PMC5503859

[3] Antonio K, Valdez MM, Mercado-Asis L, Taïeb D, Pacak K. Pheochromocytoma/paraganglioma: recent updates in genetics, biochemistry, immunohistochemistry, metabolomics, imaging and therapeutic options. Gland Surg. 2020; 9:105–23. 10.21037/gs.2019.10.25 32206603 PMC7082276

[4] Su Q, Ding Q, Zhang Z, Yang Z, Qiu Y, Li X, Mo W. Identification of genes associated with the metastasis of pheochromocytoma/paraganglioma based on weighted gene coexpression network analysis. Biomed Res Int. 2020; 2020:3876834. 10.1155/2020/3876834 32090084 PMC7025031

[5] Luo Y, Zeng G, Wu S. Identification of microenvironment-related prognostic genes in bladder cancer based on gene expression profile. Front Genet. 2019; 10:1187. 10.3389/fgene.2019.01187 31824575 PMC6883806

[6] Yoshihara K, Shahmoradgoli M, Martínez E, Vegesna R, Kim H, Torres-Garcia W, Treviño V, Shen H, Laird PW, Levine DA, Carter SL, Getz G, Stemke-Hale K, et al. Inferring tumour purity and stromal and immune cell admixture from expression data. Nat Commun. 2013; 4:2612. 10.1038/ncomms3612 24113773 PMC3826632

[7] Zhang C, Cheng W, Ren X, Wang Z, Liu X, Li G, Han S, Jiang T, Wu A. Tumor purity as an underlying key factor in glioma. Clin Cancer Res. 2017; 23:6279–91. 10.1158/1078-0432.CCR-16-2598 28754819

[8] Chen YH, Chen SH, Hou J, Ke ZB, Wu YP, Lin TT, Wei Y, Xue XY, Zheng QS, Huang JB, Xu N. Identifying hub genes of clear cell renal cell carcinoma associated with the proportion of regulatory T cells by weighted gene co-expression network analysis. Aging (Albany NY). 2019; 11:9478–91. 10.18632/aging.102397 31672930 PMC6874443

[9] Xu N, Chen SH, Lin TT, Cai H, Ke ZB, Dong RN, Huang P, Li XD, Chen YH, Zheng QS. Development and validation of hub genes for lymph node metastasis in patients with prostate cancer. J Cell Mol Med. 2020; 24:4402–14. 10.1111/jcmm.15098 32130760 PMC7176841

[10] Li X, Gao Y, Xu Z, Zhang Z, Zheng Y, Qi F. Identification of prognostic genes in adrenocortical carcinoma microenvironment based on bioinformatic methods. Cancer Med. 2020; 9:1161–72. 10.1002/cam4.2774 31856409 PMC6997077

[11] Kang JM, Lee WJ, Kim WB, Kim TY, Koh JM, Hong SJ, Huh J, Ro JY, Chi HS, Kim MS. Systemic inflammatory syndrome and hepatic inflammatory cell infiltration caused by an interleukin-6 producing pheochromocytoma. Endocr J. 2005; 52:193–98. 10.1507/endocrj.52.193 15863947

[12] Siddiqui UM, Matta S, Wessolossky MA, Haas R. Fever of unknown origin: could it be a pheochromocytoma? a case report and review of the literature. Case Rep Endocrinol. 2018; 2018:3792691. 10.1155/2018/3792691 30057828 PMC6051277

[13] Ohmura H, Uchino K, Kajitani T, Sakamoto N, Baba E. Predictive value of the modified glasgow prognostic score for the therapeutic effects of molecular-targeted drugs on advanced renal cell carcinoma. Mol Clin Oncol. 2017; 6:669–75. 10.3892/mco.2017.1205 28515920 PMC5431320

[14] Chen Z, Wang K, Lu H, Xue D, Fan M, Zhuang Q, Yin S, He X, Xu R. Systemic inflammation response index predicts prognosis in patients with clear cell renal cell carcinoma: a propensity score-matched analysis. Cancer Manag Res. 2019; 11:909–19. 10.2147/CMAR.S186976 30697081 PMC6342149

[15] Coussens LM, Werb Z. Inflammation and cancer. Nature. 2002; 420:860–67. 10.1038/nature01322 12490959 PMC2803035

[16] Karin M, Greten FR. NF-kappaB: linking inflammation and immunity to cancer development and progression. Nat Rev Immunol. 2005; 5:749–59. 10.1038/nri1703 16175180

[17] Takano S, Yokosuka O, Imazeki F, Tagawa M, Omata M. Incidence of hepatocellular carcinoma in chronic hepatitis B and C: a prospective study of 251 patients. Hepatology. 1995; 21:650–55. 10.1002/hep.1840210308 7875662

[18] Vaiphei K, Pal NS, Arora SK. Comparative analysis of HBV and HCV infection in hepatocellular carcinoma and chronic liver disease—an autopsy based study. Indian J Pathol Microbiol. 2006; 49:357–61. 17001884

[19] Sherman M. Risk of hepatocellular carcinoma in hepatitis B and prevention through treatment. Cleve Clin J Med. 2009 (Suppl 3); 76:S6–9. 10.3949/ccjm.76.s3.02 19465708

[20] Lin WW, Karin M. A cytokine-mediated link between innate immunity, inflammation, and cancer. J Clin Invest. 2007; 117:1175–83. 10.1172/JCI31537 17476347 PMC1857251

[21] Qu X, Tang Y, Hua S. Immunological approaches towards cancer and inflammation: a cross talk. Front Immunol. 2018; 9:563. 10.3389/fimmu.2018.00563 29662489 PMC5890100

[22] Liu X, Xu X, Deng W, Huang M, Wu Y, Zhou Z, Zhu K, Wang Y, Cheng X, Zhou X, Chen L, Li Y, Wang G, Fu B. CCL18 enhances migration, invasion and EMT by binding CCR8 in bladder cancer cells. Mol Med Rep. 2019; 19:1678–86. 10.3892/mmr.2018.9791 30592282 PMC6390063

[23] Chenivesse C, Tsicopoulos A. CCL18 - beyond chemotaxis. Cytokine. 2018; 109:52–56. 10.1016/j.cyto.2018.01.023 29402725

[24] Wang J, Qin Y, Zhu G, Huang D, Wei M, Li G, She L, Zhang D, Wang G, Chen X, Shen Z, Qiu Y, Wang Y, et al. High serum CCL18 predicts a poor prognosis in patients with laryngeal squamous cell carcinoma. J Cancer. 2019; 10:6910–14. 10.7150/jca.37515 31839826 PMC6909940

[25] Urquidi V, Kim J, Chang M, Dai Y, Rosser CJ, Goodison S. CCL18 in a multiplex urine-based assay for the detection of bladder cancer. PLoS One. 2012; 7:e37797. 10.1371/journal.pone.0037797 22629457 PMC3357344

[26] Yuan L, Wan J, Huang C, Liang J, Liu M, Yue C, Li L. Evaluation of serum CCL18 as a potential biomarker for ovarian cancer. Cancer Biomark. 2017; 21:97–104. 10.3233/CBM-170305 29036787 PMC13075747

[27] Ye H, Zhou Q, Zheng S, Li G, Lin Q, Wei L, Fu Z, Zhang B, Liu Y, Li Z, Chen R. Tumor-associated macrophages promote progression and the warburg effect via CCL18/NF-kB/VCAM-1 pathway in pancreatic ductal adenocarcinoma. Cell Death Dis. 2018; 9:453. 10.1038/s41419-018-0486-0 29670110 PMC5906621

[28] Zhou Z, Peng Y, Wu X, Meng S, Yu W, Zhao J, Zhang H, Wang J, Li W. CCL18 secreted from M2 macrophages promotes migration and invasion via the PI3K/Akt pathway in gallbladder cancer. Cell Oncol (Dordr). 2019; 42:81–92. 10.1007/s13402-018-0410-8 30276551 PMC12994345

[29] Wu J, Liu X, Wang Y. Predictive value of preoperative serum CCL2, CCL18, and VEGF for the patients with gastric cancer. BMC Clin Pathol. 2013; 13:15. 10.1186/1472-6890-13-15 23697837 PMC3681643

[30] Jiang X, Liu J, Li S, Jia B, Huang Z, Shen J, Luo H, Zhao J. CCL18-induced LINC00319 promotes proliferation and metastasis in oral squamous cell carcinoma via the miR-199a-5p/FZD4 axis. Cell Death Dis. 2020; 11:777. 10.1038/s41419-020-02978-w 32948745 PMC7501282

[31] Anderson KJ, Allen RL. Regulation of T-cell immunity by leucocyte immunoglobulin-like receptors: innate immune receptors for self on antigen-presenting cells. Immunology. 2009; 127:8–17. 10.1111/j.1365-2567.2009.03097.x 19368561 PMC2678177

[32] Young NT, Waller EC, Patel R, Roghanian A, Austyn JM, Trowsdale J. The inhibitory receptor LILRB1 modulates the differentiation and regulatory potential of human dendritic cells. Blood. 2008; 111:3090–96. 10.1182/blood-2007-05-089771 18094328

[33] Chui CS, Li D. Role of immunolglobulin-like transcript family receptors and their ligands in suppressor T-cell-induced dendritic cell tolerization. Hum Immunol. 2009; 70:686–91. 10.1016/j.humimm.2009.06.003 19524004

[34] Jones DC, Hewitt CR, López-Álvarez MR, Jahnke M, Russell AI, Radjabova V, Trowsdale AR, Trowsdale J. Allele-specific recognition by LILRB3 and LILRA6 of a cytokeratin 8-associated ligand on necrotic glandular epithelial cells. Oncotarget. 2016; 7:15618–31. 10.18632/oncotarget.6905 26769854 PMC4941265

[35] Hamann J, Aust G, Araç D, Engel FB, Formstone C, Fredriksson R, Hall RA, Harty BL, Kirchhoff C, Knapp B, Krishnan A, Liebscher I, Lin HH, et al. International union of basic and clinical pharmacology. Xciv. Adhesion G protein-coupled receptors. Pharmacol Rev. 2015; 67:338–67. 10.1124/pr.114.009647 25713288 PMC4394687

[36] Pan Q, Long X, Song L, Zhao D, Li X, Li D, Li M, Zhou J, Tang X, Ren H, Ding K. Transcriptome sequencing identified hub genes for hepatocellular carcinoma by weighted-gene co-expression analysis. Oncotarget. 2016; 7:38487–99. 10.18632/oncotarget.9555 27220887 PMC5122405

[37] Waddell LA, Lefevre L, Bush SJ, Raper A, Young R, Lisowski ZM, McCulloch ME, Muriuki C, Sauter KA, Clark EL, Irvine KM, Pridans C, Hope JC, Hume DA. ADGRE1 (EMR1, F4/80) is a rapidly-evolving gene expressed in mammalian monocyte-macrophages. Front Immunol. 2018; 9:2246. 10.3389/fimmu.2018.02246 30327653 PMC6174849

[38] Legrand F, Tomasevic N, Simakova O, Lee CC, Wang Z, Raffeld M, Makiya MA, Palath V, Leung J, Baer M, Yarranton G, Maric I, Bebbington C, Klion AD. The eosinophil surface receptor epidermal growth factor-like module containing mucin-like hormone receptor 1 (EMR1): a novel therapeutic target for eosinophilic disorders. J Allergy Clin Immunol. 2014; 133:1439–47. 10.1016/j.jaci.2013.11.041 24530099 PMC4113341

[39] Beacham DA, Cukierman E. Stromagenesis: the changing face of fibroblastic microenvironments during tumor progression. Semin Cancer Biol. 2005; 15:329–41. 10.1016/j.semcancer.2005.05.003 15970443

[40] Joyce JA, Pollard JW. Microenvironmental regulation of metastasis. Nat Rev Cancer. 2009; 9:239–52. 10.1038/nrc2618 19279573 PMC3251309

[41] Scheer R, Baidoshvili A, Zoidze S, Elferink MA, Berkel AE, Klaase JM, van Diest PJ. Tumor-stroma ratio as prognostic factor for survival in rectal adenocarcinoma: a retrospective cohort study. World J Gastrointest Oncol. 2017; 9:466–74. 10.4251/wjgo.v9.i12.466 29290917 PMC5740087

[42] Chang CC, Su KM, Lu KH, Lin CK, Wang PH, Li HY, Wang ML, Lin CK, Yu MH, Chang CM. Key immunological functions involved in the progression of epithelial ovarian serous carcinoma discovered by the gene ontology-based immunofunctionome analysis. Int J Mol Sci. 2018; 19:3311. 10.3390/ijms19113311 30356023 PMC6274992

[43] Kim HJ, Cantor H. CD4 T-cell subsets and tumor immunity: the helpful and the not-so-helpful. Cancer Immunol Res. 2014; 2:91–98. 10.1158/2326-6066.CIR-13-0216 24778273

[44] Chen YH, Lin TT, Wu YP, Li XD, Chen SH, Xue XY, Wei Y, Zheng QS, Huang JB, Xu N. Identification of key genes and pathways in seminoma by bioinformatics analysis. Onco Targets Ther. 2019; 12:3683–93. 10.2147/OTT.S199115 31190870 PMC6526170

[45] Yip AM, Horvath S. Gene network interconnectedness and the generalized topological overlap measure. BMC Bioinformatics. 2007; 8:22. 10.1186/1471-2105-8-22 17250769 PMC1797055

[46] Zhou Y, Zhou B, Pache L, Chang M, Khodabakhshi AH, Tanaseichuk O, Benner C, Chanda SK. Metascape provides a biologist-oriented resource for the analysis of systems-level datasets. Nat Commun. 2019; 10:1523. 10.1038/s41467-019-09234-6 30944313 PMC6447622

[47] Szklarczyk D, Franceschini A, Wyder S, Forslund K, Heller D, Huerta-Cepas J, Simonovic M, Roth A, Santos A, Tsafou KP, Kuhn M, Bork P, Jensen LJ, von Mering C. STRING v10: protein-protein interaction networks, integrated over the tree of life. Nucleic Acids Res. 2015; 43:D447–52. 10.1093/nar/gku1003 25352553 PMC4383874

[48] Shannon P, Markiel A, Ozier O, Baliga NS, Wang JT, Ramage D, Amin N, Schwikowski B, Ideker T. Cytoscape: a software environment for integrated models of biomolecular interaction networks. Genome Res. 2003; 13:2498–504. 10.1101/gr.1239303 14597658 PMC403769

